# Enhanced GATA4 expression in senescent systemic lupus erythematosus monocytes promotes high levels of IFNα production

**DOI:** 10.3389/fimmu.2024.1320444

**Published:** 2024-03-28

**Authors:** Taiga Kuga, Asako Chiba, Goh Murayama, Kosuke Hosomi, Tomoya Nakagawa, Yoshiyuki Yahagi, Daisuke Noto, Makio Kusaoi, Fuminori Kawano, Ken Yamaji, Naoto Tamura, Sachiko Miyake

**Affiliations:** ^1^ Department of Immunology, Juntendo University Faculty of Medicine, Bunkyo-ku, Tokyo, Japan; ^2^ Department of Internal Medicine and Rheumatology, Juntendo University Faculty of Medicine, Bunkyo-ku, Tokyo, Japan; ^3^ Graduate School of Health Sciences, Matsumoto University, Matsumoto, Nagano, Japan

**Keywords:** SLE, monocyte, interferon, cellular senescence, GATA4

## Abstract

Enhanced interferon α (IFNα) production has been implicated in the pathogenesis of systemic lupus erythematosus (SLE). We previously reported IFNα production by monocytes upon activation of the stimulator of IFN genes (STING) pathway was enhanced in patients with SLE. We investigated the mechanism of enhanced IFNα production in SLE monocytes. Monocytes enriched from the peripheral blood of SLE patients and healthy controls (HC) were stimulated with 2′3′-cyclic GAMP (2′3′-cGAMP), a ligand of STING. IFNα positive/negative cells were FACS-sorted for RNA-sequencing analysis. Gene expression in untreated and 2′3′-cGAMP-stimulated SLE and HC monocytes was quantified by real-time PCR. The effect of GATA binding protein 4 (GATA4) on IFNα production was investigated by overexpressing GATA4 in monocytic U937 cells by vector transfection. Chromatin immunoprecipitation was performed to identify GATA4 binding target genes in U937 cells stimulated with 2′3′-cGAMP. Differentially expressed gene analysis of cGAS-STING stimulated SLE and HC monocytes revealed the enrichment of gene sets related to cellular senescence in SLE. CDKN2A, a marker gene of cellular senescence, was upregulated in SLE monocytes at steady state, and its expression was further enhanced upon STING stimulation. GATA4 expression was upregulated in IFNα-positive SLE monocytes. Overexpression of GATA4 enhanced IFNα production in U937 cells. GATA4 bound to the enhancer region of IFIT family genes and promoted the expressions of IFIT1, IFIT2, and IFIT3, which promote type I IFN induction. SLE monocytes with accelerated cellular senescence produced high levels of IFNα related to GATA4 expression upon activation of the cGAS-STING pathway.

## 1 Introduction

Type I interferons (Type I IFNs) including IFNα, IFNβ, and IFNω are overexpressed in systemic lupus erythematosus (SLE) (1, 2). Serum levels of type I IFNs and IFN-inducible genes in peripheral blood mononuclear cells (PBMCs) were elevated and associated with disease activity in SLE (3, 4). Anifrolmab, an antagonistic human monoclonal antibody targeting the IFNα receptor 1, was shown to be effective and recently approved for the treatment of SLE (5), highlighting the importance of regulating the type I IFN pathway to suppress the pathological process of SLE.

Type I IFNs control innate and adaptive immunity in response to pathogenic infection (6). Although most nucleated cells produce type I IFNs via the detection of pathogen-associated molecular patterns by nucleic acid sensors upon pathogenic infection (6), plasmacytoid dendritic cells (pDCs) are a major producer of IFNα (6) through the Toll-like receptor (TLR) 7 and TLR9 signaling pathways (7). We previously reported that the enhanced IFNα-producing capacity of pDCs upon TLR7 agonist stimulation correlated with disease activity and serum IFNα levels in SLE (8).

The cyclic GMP-AMP synthase (cGAS)-stimulator of IFN genes (STING) pathway is an important cytoplasmic DNA recognition pathway. cGAS recognizes double-stranded DNA and produces 2′3′-cyclic GAMP (2′3′-cGAMP), which binds to STING. Activated STING translocates from the endoplasmic reticulum to the Golgi where it triggers the activation of TBK1 and IRF3, leading to the induction of type I IFNs. Although cGAS-STING activation is typically induced by DNA derived from pathogenic microbes or viruses, excessive self-DNA derived from apoptosis-derived membrane vesicles (9), neutrophil extracellular traps (NETs) (10), and mitochondrial DNA (11, 12) shed into the cytoplasm are increased in SLE and activate the cGAS-STING pathway. Previously, we reported that when human PBMCs were stimulated with 2′3′-cGAMP, monocytes but not pDCs, were the major producers of IFNα (13). In addition, monocytes from SLE patients had enhanced IFNα production upon activation of the STING pathway, and this was positively correlated with SLE disease activity (13). The enhanced IFNα production was associated with increased STING expression in SLE monocytes. Because IFNα exposure increased STING expression in monocytes from healthy individuals, SLE monocytes may have obtained the capacity to produce high levels of IFNα due to prior *in vivo* exposure to IFNα. However, it is not known whether a cell-intrinsic mechanism is responsible for the increased production of IFNα by SLE monocytes.

In the present study, we elucidated the mechanism of enhanced IFNα production in SLE monocytes. The RNA-sequencing (RNA-seq) analysis of monocytes activated via the cGAS-STING pathway revealed that genes upregulated in SLE monocytes were enriched for gene sets related to cellular senescence, a state of cell cycle arrest that can be induced by various cellular stressors including DNA damage (14). Moreover, IFNα producing SLE monocytes had an upregulated expression of GATA binding protein 4 (GATA4), a transcription factor that is a critical regulator of the senescence-associated secretory phenotype (SASP) in cellular senescence (15, 16). GATA4 overexpression increased cGAS-STING-mediated IFNα induction by monocytic U937 cells. GATA4 promoted the expressions of interferon-induced protein with tetratricopeptide repeats (IFIT) family genes, which encode proteins essential for antiviral immune responses (17) and enhance the induction of Type I IFNs through various mechanisms (18, 19). Our results showed that SLE monocytes with a cellular senescence phenotype had an enhanced IFNα production capacity related to GATA4 expression upon cGAS-STING stimulation. When a significant increase occurs in cytoplasmic DNA load in patients with SLE, such as during flares, these mechanisms underlying the increased reactivity to cGAS-STING may contribute to increased disease activity.

## 2 Materials and methods

### 2.1 Human samples

Peripheral blood was drawn from SLE patients and healthy controls (HCs) after obtaining informed consent in accordance with the local ethical committee guidelines of Juntendo University. SLE was diagnosed according to the American College of Rheumatology criteria for SLE. Disease activity was assessed by the SLE Disease Activity Index 2000 (SLEDAI-2K). HCs had no history of autoimmune disease and had never received immunosuppressive therapy. Informed consent was obtained from all patients with SLE and HCs according to the ethical guidelines for human subject research. The RNA-seq samples included five patients diagnosed with SLE [three women, two men, median age (interquartile range) 30.0 years (29.0–46.0)], and five healthy controls (HC) [three women, two men, median age (interquartile range) 41.0 years (30.0–48.0)]. Monocytes for reverse transcription-quantitative PCR (RT-qPCR) analysis were obtained from 10 patients diagnosed with SLE [9 women, 1 man, median age (interquartile range) 45.5 years (35.3–50.0)], and 6 healthy controls (HC) [5 women, 1 man, median age (interquartile range) 37.5 years (30.5–48.8)]. For senescence-associated β-galactosidase (SA-β-Gal) analysis, PBMCs were obtained from 10 patients diagnosed with SLE [8 women, 2 men, median age (interquartile range) 47.0 years (37.0–60.0)], and 6 healthy controls (HC) [5 women, 1 man, median age (interquartile range) 42.0 years (31.3–53.3)]. The characteristics of patients are presented in **Supplementary Tables S1**–**S3**. Fresh PBMCs were isolated from whole blood by density-gradient centrifugation using BD Vacutainer CPT Mononuclear Cell Preparation Tubes with sodium heparin (BD Biosciences, New Jersey, USA).

### 2.2 Cell culture

Monocytes were enriched from PBMCs using CD14 MicroBeads (Miltenyi Biotec, Bergisch Gladbach, Germany) and magnetic-activated cell sorting (MACS), and then cultured in 96-well flat-bottomed plates in RPMI 1640 medium (Thermo Fisher Scientific, Waltham, MA, USA) containing 10% fetal bovine serum, 2 mM L-glutamine, 50 U/ml penicillin, 50 µg/ml streptomycin, and 55 µM 2-mercaptoethanol (all from Thermo Fisher Scientific) at 37°C in a 5% CO_2_ incubator. Monocytes were stimulated with recombinant human IL-3 (200 ng/ml; PeproTech, Rocky Hill, NJ, USA) and 2’3’-cGAMP (50 µg/ml; *In vivo*Gen, San Diego, CA, USA) for 5 h, except for the immunofluorescence experiment where monocytes were stimulated for 3 h. HC monocytes used for RNA-seq experiments were pretreated with IFNα (100 U/ml) (R&D Systems, Minneapolis, MN, USA) for 18 h prior to stimulation with 2’3’-cGAMP.

### 2.3 Cell sorting and sample preparation for RNA-seq

MACS-isolated monocytes from SLE and HC individuals were stimulated with 2’3’-cGAMP. HC monocytes were pretreated with IFNα (100 U/ml) (R&D Systems) for 18 h prior to stimulation with 2’3’-cGAMP. IFNα-producing monocytes stimulated with 2’3’-cGAMP were detected using an IFNα Secretion Assay kit (PE) (Miltenyi Biotec) following the manufacturer’s instructions. Cell surface staining was performed with antibodies against CD3 (3.9, FITC, BioLegend, San Diego, CA, USA), CD123 (6H6, FITC, BioLegend), CD3 (UCHT1, PerCP/Cyanine5.5, BioLegend), CD19 (HIB19, PerCP/Cyanine5.5, BioLegend), CD56 (HCD56, PerCP/Cyanine5.5, BioLegend), and CD14 (M5E2, Brilliant Violet 421, BioLegend). 7-AAD (BD Biosciences) was added for dead cell exclusion and samples were sorted by BD FACS Aria III (BD Biosciences). A representative FACS plot of IFNα positive/negative monocytes from a SLE patient is shown in **Supplementary Figure S1**. Live CD14^+^ cells negative for lineage markers (CD3, CD19, CD56, CD11c, CD123) were sorted according to IFNα secretion.

### 2.4 RNA-seq library preparation

Total RNA was extracted from FACS-sorted cells using an RNeasy mini Kit (Qiagen, Hilden, Germany). The sequencing library was constructed by following the Ovation SoLo RNA-seq system Human kit (NuGEN Technologies, San Carlos, CA, USA), using 5 ng of total RNA to generate cDNA. The cDNA libraries were sequenced by 50-base single-read sequencing on an Illumina NovaSeq 6000 system (Illumina, San Diego, CA, USA). The sequencing run and base call analysis were performed according to the Ovation SoLo RNA-Seq system Human M01406 v4. Raw sequence data were generated with processing by CASAVA-1.8.4 with version RTA 1.17.20.0.

### 2.5 RNA-seq analysis

Fastq files were processed for quality control, adapter trimming, and quality filtering using fastp. Reads were mapped to the GRCh38 human genome with HISAT2 (v2.2.0), and the uniquely mapped reads were counted by FeatureCounts (v2.0.1). Read count data were analyzed further by iDEP.91 (http://bioinformatics.sdstate.edu/idep90/). Genes were filtered out if they failed to achieve < 0.5 Counts per million (CPM) in at least one library, and count data were transformed using EdgeR [log2(CPM+c)] (pseudocount c = 4). Differentially expressed genes (DEGs) identified by DESeq2 were defined as genes with a fold change > 2 or < −2, and an adjusted P-value <.05. A schematic diagram of sample selection for DGE analysis is provided in **Supplementary Figure S2**. The same methods were applied for the analysis of publicly-available RNA-seq dataset (PRJNA:392602). For the differential gene expression analysis of SLE monocytes and HC monocytes, IFNα-positive samples and IFNα-negative samples obtained from each individual were averaged. For individual gene expression plots derived from RNA-seq, two-way ANOVA and Tukey’s multiple comparison test were performed. Enrichment analysis using Metascape software (http://metascape.org/gp/index.html#/main/step1) was conducted based on databases including Gene Onthology, Reactome, and CORUM. A gene set enrichment analysis (GSEA) software tool was downloaded from the Broad Institute website (https://www.gsea-msigdb.org/gsea/index.jsp) as a Java desktop application. GSEA was performed using the default settings with weighted enrichment statics and Signal2Noise metrics for ranking genes except for analyses in which GATA4 (ENSG00000136574) was assigned as a gene used for phenotype labels and Pearson metric for ranking genes in IFNα-positive and IFNα-negative SLE monocytes.

### 2.6 RT-qPCR

Total RNA was isolated using RNeasy Mini kits (Qiagen) according to the manufacturer’s instructions. Reverse transcription was performed using the ReverTra Ace qPCR RT Master Mix with gDNA Remover (TOYOBO, Osaka, Japan) according to the manufacturer’s instructions. Real-time PCR was performed in a 7500 Real-Time PCR System (Applied Biosystems, Foster City, CA, USA) using a Power SYBR green PCR master mix (Applied Biosystems) according to the manufacturer’s instructions. For each sample tested, the levels of the indicated mRNA were normalized to the levels of GAPDH mRNA. The 2^-ΔΔCT^ method was used to calculate the relative mRNA expression. The sequences of the primers used in this study are listed in **Supplementary Table S4**.

### 2.7 Confocal microscopy analysis

MACS-isolated monocytes from SLE patients and HC individuals were stimulated with 2’3’-cGAMP for 3 h. Monocytes freshly isolated or stimulated with 2’3’-cGAMP were spun onto a microscope slide using Thermo Shandon Cytospin 4 (Thermo Fisher Scientific). Monocytes were fixed with 4% paraformaldehyde and then permeabilized with 0.1% Triton X-100 in PBS. Nonspecific background staining was prevented by incubation with 5% bovine serum albumin in PBS. Cells were incubated overnight at 4°C with the following primary antibodies: anti-GATA4 (1:100, Proteintech, Rosemont, IL, USA, Cat. #19530) and anti-p16 (1:100, Cell Signaling Technology, Danvers, MA, USA, Cat. #18769), and then washed and incubated for 1 h at room temperature with a secondary antibody, Alexa 647 anti-rabbit IgG (1:1000, Cell Signaling Technology, Cat. #4414). All samples were visualized using a Leica TCS SP5 confocal scanning microscope (Leica, Mannheim, Germany), and images were processed using Leica LASAF software and ImageJ (http://imagej.nih.gov/ij/).

### 2.8 Senescence-associated β-galactosidase activity assay

SA-β-Gal activity was measured by flow cytometry using a SPiDER-β-Gal probe (Dojindo Molecular Technologies, Kumamoto, Japan, Cat. #SG02-10), according to the manufacturer’s instructions. Briefly, fresh PBMCs were harvested and pre-treated with 100 nM Bafilomycin A1 (Enzo Life Sciences, New York, NY, Cat. #BML-CM110-0100) for 1 h before incubation with 1 µM SPiDER-β-Gal for 30 min. Cells were stained using a Zombie Yellow™ Fixable Viability Kit (BioLegend, Cat. #423104) and then with combinations of the following monoclonal antibodies against CD3 (UCHT1, PerCP/Cyanine5.5, BioLegend), CD19 (HIB19, Alexa Fluor 700, BD Biosciences), CD56 (N901, APC, Beckman Coulter, Indianapolis, IN, USA), and CD14 (M5E2, Brilliant Violet 421, BioLegend) for 30 min in ice. Monocytes were identified as CD3^-^ CD19^-^ CD56^-^ CD14^+^. Data were acquired on a FACS LSR Fortessa (BD Biosciences) and analyzed using FlowJo software (TreeStar Inc., Ashland, OR, USA).

### 2.9 U937 cell culture and transfection

U937 cells were grown in RPMI 1640 medium containing 10% fetal bovine serum, 2 mM L-glutamine, 50 U/ml penicillin, 50 µg/ml streptomycin, and 55 µM 2-mercaptoethanol (all from Thermo Fisher Scientific) at 37°C in a 5% CO_2_ incubator. A human GATA4 overexpression vector (pRP[Exp]-EGFP-CMV>hGATA4[NM_001308093.3]) and empty vector control (pRP[Exp]-EGFP-CMV>ORF_Stuffer) were designed and purchased from Vector Builder (Chicago, IL, USA). U937 cells were transfected with the GATA4 overexpression vector or empty vector control for 24 h and then stimulated with 2’3’-cGAMP (0.5 µg/ml) for 6 h. The lipofection agent DOTAP (Roche, Basel, Switzerland) was used for the transfection of the plasmid vectors and stimulation with 2’3’-cGAMP according to the manufacturer’s instructions. For chromatin immunoprecipitation (ChIP)-seq and ChIP-PCR, U937 cells were stimulated with 2’3’-cGAMP (5 µg/ml) for 4 h.

### 2.10 ChIP-seq and ChIP-qPCR

ChIP assays were performed using a CUT&RUN Assay Kit (Cell Signaling Technology) according to the manufacturer’s instructions. All the reagents other than antibodies were included in the kit. Briefly, U937 cells were stimulated with 2’3’-cGAMP (5 µg/ml) or were unstimulated for 4 h, washed, and then 100,000 cells were bound to activated concanavalin A magnetic beads and permeabilized. Cells were then incubated with anti-GATA4 antibody (Invitrogen, Waltham, MA, USA, Cat. #MA5-15532) or anti-mouse IgG (Calbiochem, San Diego, CA, USA, Cat. #N103-100UG) for 3 h at 4°C. After washing, cells were incubated with pAG-Micrococcal nuclease (MNase) Enzyme, and then the digestive reaction was activated by adding calcium chloride for 30 min at 4°C. The reaction was stopped by Stop Buffer and released chromatin fragments were purified by DNA Purification Buffer and Spin Columns (Cell Signaling Technology). Input DNA was prepared by the DNA fragmentation protocol using MNase and purified by Spin Columns. For ChIP-seq, libraries were generated using the TruSeq DNA sample prep Kit (Illumina) and sequenced on the NovaSeq6000. Fastq files were processed for quality control, adapter trimming, and quality filtering using the fastp program. Reads were mapped to the GRCh38 human genome using HISAT2. To analyze the histone modification status and GATA4 occupancy of the genomic regions surrounding IFIT1B and IFIT1, publicly-available ChIP-seq datasets aligned to hg38, which were processed in the ChIP-Atlas database (http://chip-atlas.org/), were obtained as BigWig files and visualized using the genome viewer IGV (https://igv.org/). Peak calling was performed using a hypergeometric optimization of Motif Enrichment (Homer, version 4.11). The publicly-available ChIP-seq datasets used in **Supplementary Figure S3A** are listed in **Supplementary Table S5**. For ChIP-qPCR, purified DNA was quantified by RT-qPCR using primers targeting the genomic regions upstream of IFIT1B and IFIT1. The design and sequences of the primers used in this analysis are shown in **Supplementary Figure S3B**.

### 2.11 Statistical analysis

All data were analyzed using GraphPad Prism (GraphPad, San Diego, CA, USA). Two-sided unpaired *t*-tests or two-tailed Mann–Whitney *U*-tests were used for comparisons between two groups. Multiparameter analyses of cell-based studies were performed by two-way analysis of variance (ANOVA) followed by Tukey’s *post hoc* test for multiple comparisons. Correlations between genes were calculated by pearson's correlation coefficient. Statistical significance was defined as ****p < 0.0001, ***p < 0.001, **p < 0.01, and *p < 0.05. Statistical parameters including the exact value of n, precision measures, and statistical significance are reported in the figures and figure legends.

## 3 Results

### 3.1 A cellular senescence phenotype is observed in SLE monocytes

**Figure 1:**
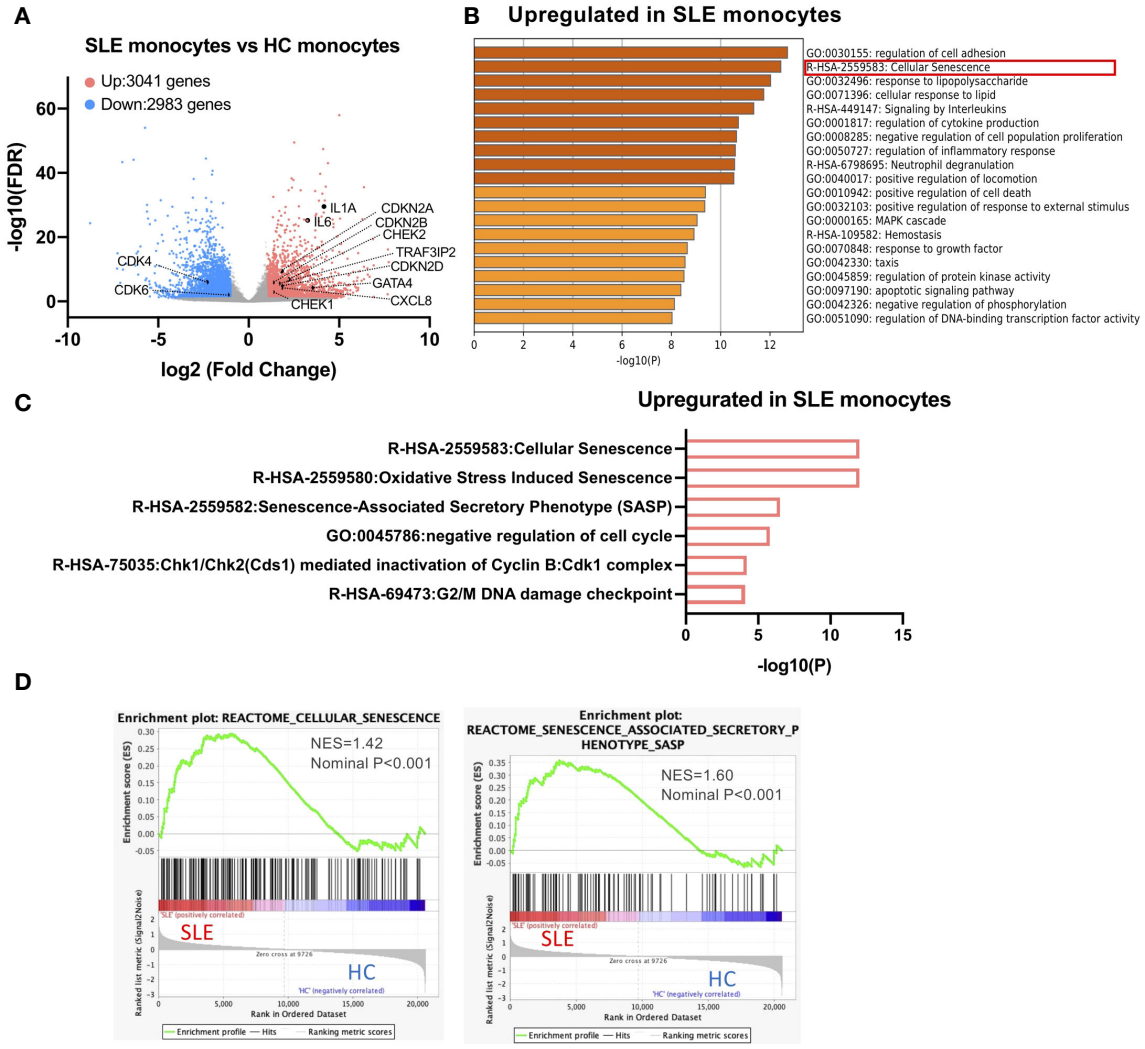
A cellular senescence phenotype is observed in SLE monocytes. RNA-sequencing (RNA-seq) was performed in SLE and HC monocytes stimulated with 2′3′-cGAMP for 5 h. HC monocytes were pretreated with IFNα (100 U/ml) for 18 h prior to the stimulation with 2′3′-cGAMP. **(A)** A volcano plot comparing the false discovery rate (FDR) and fold-change of total gene expression data from SLE monocytes and HC monocytes. **(B, C)** Enrichment analysis of differentially expressed genes (DEGs) upregulated in SLE monocytes was performed using Metascape. **(B)** Bar graph of the top 20 non-redundant enrichment clusters across DEGs upregulated in SLE monocytes, ranked by p-value. **(C)** Bar graph of enriched gene sets related to cellular senescence across DEGs upregulated in SLE monocytes. **(D)** Gene set enrichment analysis (GSEA) plot showing the enrichment of “Cellular senescence (R-HSA-2559583)”, and “Senescence-Associated Secretory Phenotype (SASP) (R-HSA-2559582)” in SLE monocytes.

To reveal the transcriptional characteristics of STING-activated SLE monocytes, we performed the RNA-seq analysis of SLE and HC monocytes stimulated with 2′3′-cGAMP, a STING ligand. Previously, we showed that although only a small fraction of HC monocytes produced IFNα, more than 10% of SLE monocytes produced IFNα, and IFNα treatment increased STING expression and IFNα production by HC monocytes (13). To compare SLE and HC monocytes, we chose to pre-treat HC monocytes with IFNα for two reasons. First, our goal was to elucidate cell intrinsic factors responsible for enhanced IFNα production by SLE monocytes rather than attributing it solely to exposure to IFNα *in vivo*. Second, we wanted to overcome the technical difficulty of obtaining a sufficient amount of RNA from HC IFNα-producing monocytes for RNA-seq analysis due to the small number of these cells. To characterize the general features of SLE monocytes after STING activation, we performed differential gene expression analysis between STING-activated SLE and HC monocytes (schematic diagram is shown in **Supplementary Figure S2**). Compared with HC monocytes, 3041 genes were upregulated and 2983 genes were downregulated in SLE monocytes (**Figure 1A**). Enrichment analysis of the genes differentially upregulated in SLE monocytes revealed a highly enriched gene cluster for “Cellular senescence (R-HSA-2559583)” (**Figure 1B**). This cluster includes gene sets such as “Senescence-Associated Secretory Phenotype (SASP) (R-HSA-2559582)”, “Chk1/Chk2(Cds1) mediated inactivation of Cyclin B:Cdk1 complex (R-HSA-75035)”, and “G2/M DNA damage checkpoint (R-HSA-69473)”, indicating the presence of enhanced DNA damage and the DNA damage response (DDR), as well as subsequent senescence induction in SLE monocytes (**Figures 1B, C**). The differentially upregulated genes in SLE monocytes included canonical senescence-related genes such as CDKN2A, CDKN2B, and CDKN2D, as well as DNA damage-related genes such as CHEK1 and CHEK2. However, the differentially downregulated genes in SLE monocytes included genes such as CDK4 and CDK6 (**Figure 1A**), which have a positive role in cell cycle progression and are directly inhibited by CDKN2A. Gene set enrichment analysis (GSEA) also showed enrichment of genes for “Cellular senescence (R-HSA-2559583)” and “Senescence-Associated Secretory Phenotype (SASP) (R-HSA-2559582)” in SLE monocytes (**Figure 1D**), suggesting enhanced cellular senescence in STING pathway-activated SLE monocytes.

### 3.2 GATA4 is upregulated in IFNα-producing SLE monocytes

**Figure 2:**
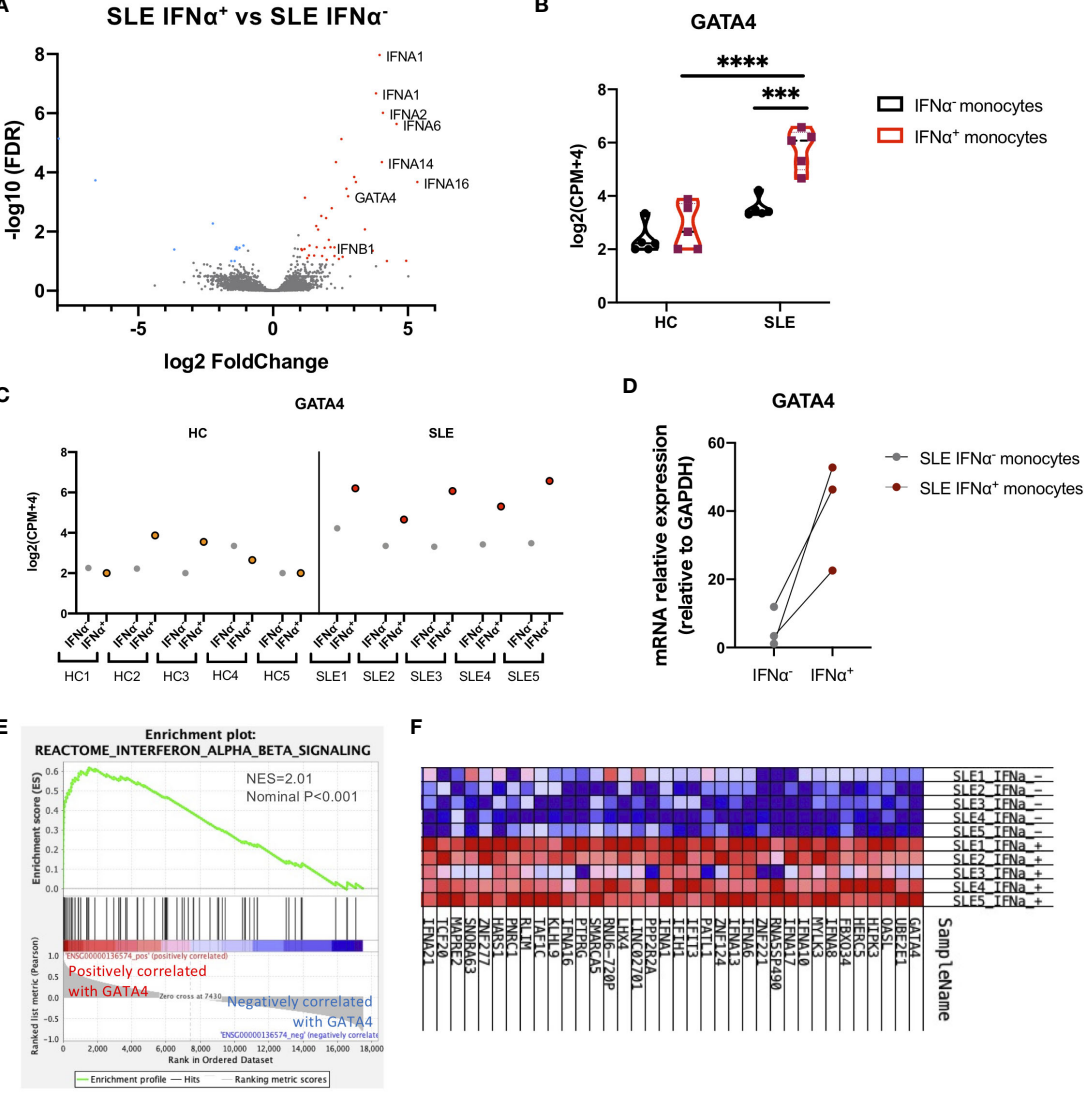
GATA4 is upregulated in IFNα-producing SLE monocytes. RNA-seq analysis of IFNα positive/negative SLE and HC monocytes in the experiments of **Figure 1**. **(A)** A volcano plot comparing the FDR and fold-change of total gene expression in RNA-seq data of IFNα^+^ and IFNα^−^ SLE monocytes. **(B)** A violin plot showing GATA4 gene expression levels in RNA-seq data of IFNα^+^ and IFNα^−^ monocytes from SLE or HC. Each dot indicates the log2 transformed gene expression level of GATA4 (mean ± SEM, two-way analysis of variance (ANOVA) and Tukey’s multiple comparisons test. ***P<0.001 and ****P<0.0001). **(C)** GATA4 gene expression levels in RNA-seq data from individual samples are plotted. IFNα^−^ = IFNα^−^ monocytes, IFNα^+^ = IFNα^+^ monocytes. **(D)** Gene expression of mRNA GATA4 was quantified by real-time reverse transcription-quantitative PCR (RT-qPCR) in IFNα^−^ and IFNα^+^ SLE monocytes. **(E, F)** GSEA analysis using GATA4 (ENSG00000136574) as a phenotype gene was performed in RNA-seq data of IFNα^+^ and IFNα^−^ SLE monocytes. **(E)** GSEA plot showing the enrichment of “Interferon Alpha Beta Signaling (R-HSA-909733)” in genes that have a positive correlation with GATA4 expression. **(F)** Expression profiles of genes with expressions that were positively correlated with GATA4 expression. Heatmap red-blue color intensity indicates the expression level of genes (red, high; white, average; blue, negative).

To investigate the mechanisms through which IFNα production is enhanced in SLE monocytes, gene expression profiles were compared between IFNα positive and negative SLE monocytes after STING activation. Fifty-two genes including type-I IFNs were differentially expressed in IFNα-positive SLE monocytes (**Figure 2A**). We found that GATA4, a key transcription factor that is an inducer of SASP, was upregulated in IFNα-producing SLE monocytes. GATA4 expression was significantly higher in IFNα^+^ SLE monocytes compared with IFNα^−^ SLE monocytes, and IFNα^+^ and IFNα^−^ HC monocytes (**Figures 2B, C**). RT-qPCR analysis confirmed that GATA4 mRNA expression was higher in IFNα^+^ monocytes compared with IFNα^−^ monocytes in SLE (**Figure 2D**). Consistent with this result, another GSEA analysis aimed at identifying gene sets that are related to GATA4 expression patterns revealed that the expressions of gene sets related to type I IFNs such as “Interferon Alpha Beta Signaling (R-HSA-909733)” were correlated with that of GATA4, suggesting the involvement of GATA4 in the production of IFNα (**Figures 2E, F**).

### 3.3 GATA4 induced by cGAS-STING stimulation is upregulated in senescent SLE monocytes

**Figure 3:**
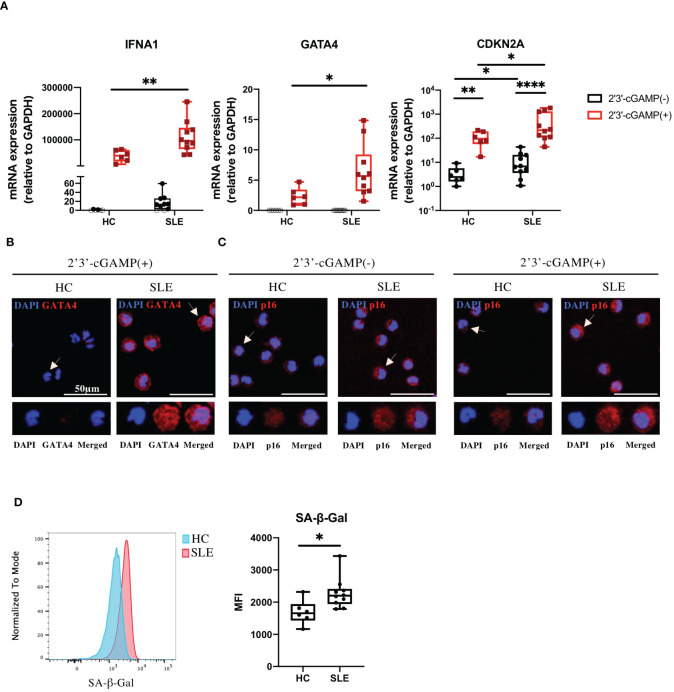
GATA4 induced by cGAS-STING stimulation is upregulated in senescent SLE monocytes. **(A)** SLE or HC monocytes were untreated or stimulated with 2′3′-cGAMP. IFNA1, GATA4, and CDKN2A expressions were quantified by RT-qPCR. The 2^-ΔΔCT^ method was used to calculate the relative gene expression, with normalization to GAPDH as the internal control. Open circles indicate no amplification by RT-qPCR. (The boxplots indicate the median, 25th percentile, and 75th percentile, and whiskers indicate the minimum and maximum values. *P<0.05, **P<0.01, and ****P<0.0001 by the Mann-Whitney *U-*test). **(B, C)** Immunofluorescence images showing GATA4 (red) staining of monocytes stimulated with 2′3′-cGAMP **(B)** or p16 (red) staining of monocytes, with and without 2′3′-cGAMP stimulation **(C)**. Cell nuclei were stained with DAPI. Representative images from HC and SLE are shown. The figure shows merged images in the upper panel, with magnified views of the indicated cells (white arrow) in the lower panel. Images correspond to DAPI (blue), GATA4 or p16 (red), and merged channels. **(D)** SA-β-Gal activity in HC and SLE monocytes was measured by flow cytometry. Histograms are representative examples of SA-β-Gal activity in HC and SLE monocytes. The boxplots indicate the median, 25th percentile, and 75th percentile, and whiskers indicate the minimum and maximum values. *P<0.05, by the Mann-Whitney *U-*test.

Because our RNA-seq data were generated from STING-activated monocytes, we asked whether GATA4 was constitutively upregulated in lupus monocytes. Monocytes from SLE or HC individuals were untreated or stimulated with 2′3′-cGAMP and then their gene expressions were quantified by RT-qPCR. As expected, IFNA1 expression was induced at higher levels in stimulated monocytes from SLE patients compared with those from HC (**Figure 3A**). GATA4 expression was not detected in unstimulated monocytes from HC and SLE, and was induced at significantly higher levels in SLE monocytes after STING stimulation (**Figure 3A**). We found that CDKN2A expression was upregulated in unstimulated monocytes from SLE patients, and STING stimulation induced CDKN2A at higher levels in SLE monocytes than in HC monocytes (**Figure 3A**). To verify the mRNA data at the protein level, the protein expressions of GATA4 and p16, the latter encoded by CDKN2A, were examined in HC and SLE monocytes. GATA4 protein expression after STING stimulation was higher in SLE monocytes than in HC monocytes (**Figure 3B**; **Supplementary Figure S4A**). In unstimulated monocytes, SLE monocytes expressed higher levels of p16 compared with HC monocytes (**Figure 3C**; **Supplementary Figure S4B**). After STING stimulation, the expression level of p16 was increased and higher in SLE monocytes compared with HC monocytes (**Figure 3C**; **Supplementary Figure S4B**). To confirm that the changes in CDKN2A expression in unstimulated SLE monocytes were associated with a cellular senescence phenotype, we measured the level of senescence-associated β-galactosidase (SA-β-Gal) activity in unstimulated HC and SLE monocytes by flow cytometry. The level of SA-β-Gal activity was higher in SLE monocytes (**Figure 3D**), suggesting that *ex vivo* monocytes from SLE patients exhibited the accelerated cellular senescence phenotype prior to cGAS-STING stimulation. To support these findings, we analyzed publicly available RNA-seq datasets of steady-state monocytes derived from the PBMCs of treatment-naïve SLE patients and HC controls (PRJNA:392602). Although GATA4 expression was not detected in any samples (data not shown), GSEA analysis revealed that “DNA Damage Telomere Stress Induced Senescence (R-HSA-2559586)”, and “Double-Strand Break Repair (R-HSA-5693532)” were enriched in SLE monocytes (**Supplementary Figure S4C**). These results suggest that the pre-existing cellular senescence phenotype in SLE monocytes may be enhanced by cGAS-STING stimulation, resulting in the higher expression of GATA4.

### 3.4 GATA4 is a positive regulator of cGAS-STING mediated IFNα induction

**Figure 4:**
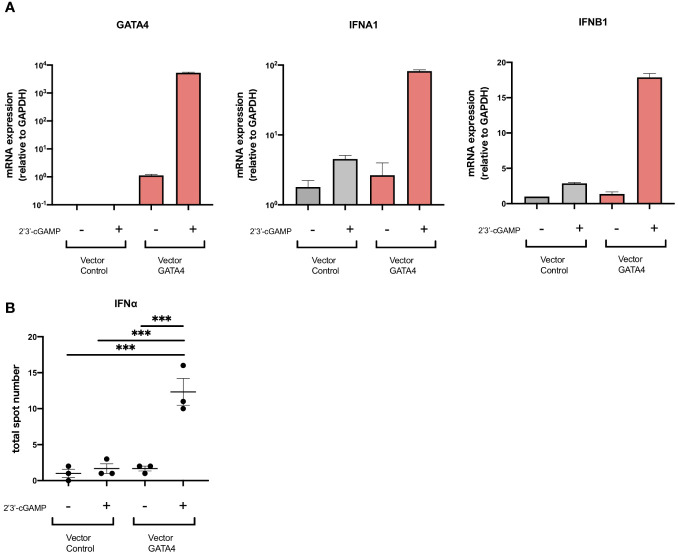
GATA4 is a positive regulator of cGAS-STING-mediated IFNα induction. **(A)** U937 cells were transfected with a GATA4 expression vector or control empty vector and then stimulated with 2′3′-cGAMP. GATA4, IFNA1, and IFNB expressions were quantified by RT-qPCR. The 2^-ΔΔCT^ method was used to calculate the relative gene expression, with normalization to GAPDH as the internal control. Results are representative of three independent experiments (mean ± SEM of triplicate RT-qPCR measurements). **(B)** U937 cells transfected with a GATA4 expression vector or control empty vector were placed into the wells of an IFNα ELISpot plate. The ELISpot plates were processed after 24 h of cell culture with 2′3′-cGAMP. Results are representative of two independent experiments (mean ± SEM, one-way ANOVA and Tukey’s multiple comparison test. ***p<0.001).

Although GATA4 has been reported to enhance cytokine production via NF-κB signaling, its involvement in the production of type I IFNs has not been demonstrated. To examine whether GATA4 plays a role in IFNα induction, we transfected U937 cells with a GATA4 expression vector or empty vector control, and then stimulated them with 2′3′-cGAMP. The overexpression of GATA4 significantly increased IFNA1 and IFNB1 expression induced by 2′3′-cGAMP stimulation (**Figure 4A**). In addition, IFNα production by U937 cells through GATA4 overexpression was confirmed at the protein level by ELISpot assay (**Figure 4B**). These results indicated that GATA4 positively regulates cGAS-STING-mediated IFNα induction.

### 3.5 GATA4 regulates IFIT family gene expression through the GATA4-bound enhancer element

**Figure 5:**
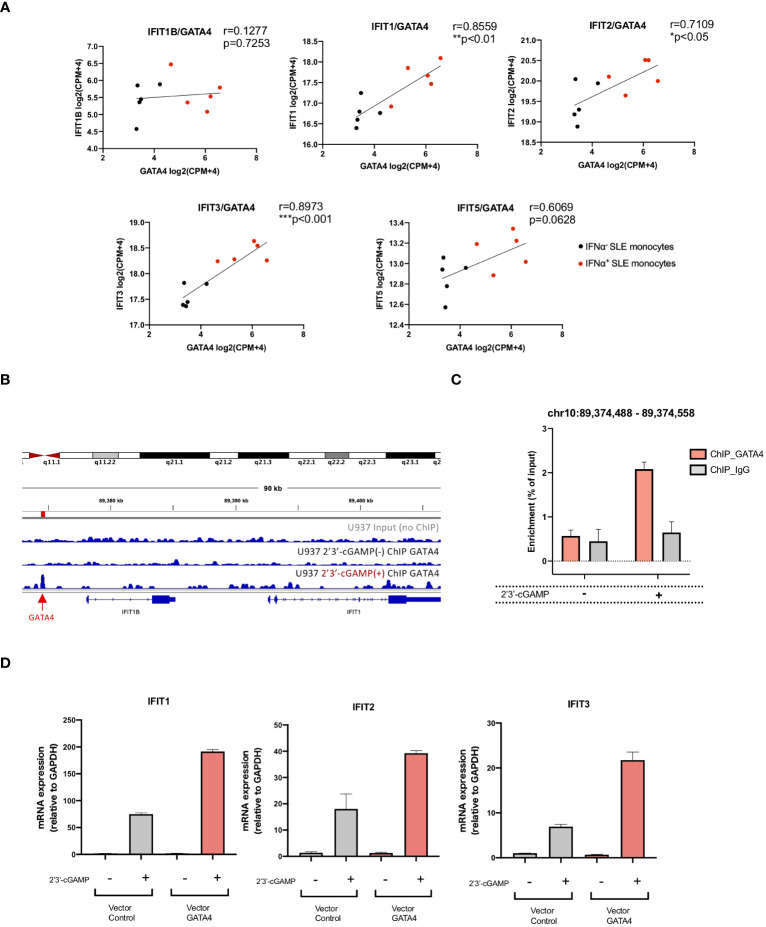
GATA4 regulates IFIT family gene expression through the GATA4-bound enhancer element. **(A)** The correlation between the gene expressions of GATA4 and IFIT family genes in RNA-seq data for IFNα^+^ monocytes and IFNα^−^ monocytes from SLE was assessed by Pearson’s correlation analysis (*P<0.05, **P<0.01, and ***P<0.001). **(B)** GATA4 chromatin immunoprecipitation (ChIP)-seq analysis in U937 cells with or without 2′3′-cGAMP stimulation was performed. Genome Browser image of the region upstream of IFIT1B and IFIT1. The arrow and red bar indicate the predicted GATA4-binding site. **(C)** GATA4 ChIP was performed in U937 cells with or without 2′3′-cGAMP stimulation. GATA4 binding at the genomic region (chr10: 89,374,488 – 89,374,558) was determined by ChIP-qPCR. Results are representative of three independent experiments (mean ± SEM of triplicate RT-qPCR measurements). **(D)** U937 cells transfected with a GATA4 expression vector or empty vector control were stimulated with 2′3′-cGAMP. Expressions of IFIT1, IFIT2, and IFIT3 were quantified by RT-qPCR. The 2^-ΔΔCT^ method was used to calculate the relative gene expression, with normalization to GAPDH as the internal control. Results are representative of three independent experiments (mean ± SEM of triplicate RT-qPCR measurements).

To identify the molecules regulated by GATA4, we examined the genes ranked according to the correlation of their expression with GATA4 expression obtained from the GSEA analysis of SLE monocyte RNA-seq data shown in **Figure 2F**. Within the top 100 genes, two were IFIT family genes, IFIT3 and IFIT1, which were previously reported to enhance the induction of type I IFNs (18, 19) (**Supplementary Data 1**). Consistent with these results, Pearson’s correlation coefficient analysis demonstrated the expression levels of IFIT1, IFIT2, and IFIT3 correlated with GATA4 expression in SLE monocytes (**Figure 5A**).

To investigate whether GATA4 binds to genomic regions that regulate the expressions of IFITs, we referenced publicly available ChIP-seq analysis of GATA4 binding signal (GSE:51705 and GSE:92491) and histone occupancy in lipopolysaccharide (LPS)-stimulated human monocytes (GSE:85245). Although no GATA4 binding site was observed in the upstream promoter regions of the IFIT genes, two regions of GATA4 binding sites upstream of IFIT1B were enriched only for H3K4me1, demonstrating the enhancer profile of these regions (**Supplementary Figure S3A**). To validate these findings, we performed a ChIP-seq analysis of GATA4 occupancy in U937 cells. However, peak calling using HOMER did not identify a GATA4-bound peak in the genomic region surrounding the IFIT genes (data not shown). Because transcription factor binding sites induced by stimulation with factors such as LPS have been reported to have lower signal intensity (20), we suspected that a weak GATA4 binding signal induced in the region approximately 3000 bp upstream of IFIT1B in U937 cells upon 2′3′-cGAMP stimulation might be significant (**Figure 5B**). ChIP-qPCR analysis confirmed that the GATA4 binding in this region was enriched in U937 cells stimulated with 2′3′-cGAMP (**Figure 5C**). To determine whether this region regulated the gene expressions of IFIT genes, we searched for the GATA4 binding region in the HACER database (https://bioinfo.vanderbilt.edu/AE/HACER/index.html), which catalogs enhancers within transcriptional regulatory networks by integrating chromatin interaction data such as GRO-seq, PRO-seq, and CAGE. The search result showed that the region overlapped with an integrated enhancer that regulates the transcription of IFIT1 and IFIT2 (**Supplementary Figure S3C**). Additionally, the GATA4 binding region (chr10:89374325-89374685; GRCh38/hg38 assembly) was searched for in the human super-enhancers database SEdb2.0 (https://bio.liclab.net/sedb/index.php). We found that the region was included in the super-enhancer (SE_02_045200796), which regulates the expression of IFIT genes including IFIT1, IFIT2, and IFIT3 (**Supplementary Figure S3D**). The overexpression of GATA4 resulted in the enhanced induction of IFIT1, IFIT2, and IFIT3 upon 2′3′-cGAMP stimulation (**Figure 5D**). These results suggest that GATA4 induced upon STING activation may translocate to the nucleus and bind to the enhancer region of IFIT family genes, resulting in enhanced IFNα production.

## 4 Discussion

IFNα has a critical role in the pathogenesis of lupus (1, 2). Elevated IFNα levels during periods of remission predicted flares in SLE patients (21, 22), and elevated interferon-stimulated gene expressions preceded flares in SLE patients (23). These findings suggest that the upregulation of IFNα may be involved in the occurrence of flares in SLE patients. An excessive DNA load due to infection (6) or exposure to ultraviolet light exposure (24) was shown to lead to the activation of cytosolic nucleic acid receptors, resulting in the activation of the cGAS-STING pathway and IFNα production. Because our previous study demonstrated that IFNα production upon STING stimulation associated with disease activity was observed in monocytes, it is important to elucidate the mechanisms underlying the enhanced IFNα production in monocytes to prevent disease flares. In this study, we demonstrated that SLE monocytes had a cellular senescence phenotype, and identified GATA4 as a key transcription factor related to the enhanced production of IFNα by SLE monocytes.

Cellular senescence was first reported when Hayflick and Moorhead observed that primary human cells had a maximum number of cell proliferations *in vitro* (25). The benefit of cellular senescence is to prevent the unfavorable proliferation of damaged cells and cancerous cells (26, 27). However, recent studies have shown that cellular senescence is involved in chronic inflammation (28). Here, we provide evidence that monocytes from SLE patients exhibited a cellular senescence phenotype, as evidenced by CDKN2A upregulation and increased SA-β-Gal activity. Persistent activation of the DDR pathway can trigger cellular senescence (14, 29). The accumulation of damaged DNA translocated to the cytoplasm activates nucleic acid receptors including cGAS (30). This leads to activation of the STING pathway, which results in the induction of cellular senescence and production of proinflammatory cytokines (30). Our transcriptome analysis revealed that gene sets related to DNA damage and DDR were enhanced in SLE monocytes under steady-state conditions and after STING activation. Consistent with our study, an increased baseline double-stranded break, measured by phospho-H2AX levels, was reported in monocytes from SLE patients (31), suggesting that double-stranded breaks and subsequent DDR may be important for cellular senescence in SLE monocytes.

The causes of DNA damage and DDR in SLE remain elusive. Impaired DNA damage repair has been proposed as a possible explanation, supported by the presence of mutations in related genes and findings from *in vitro* experiments (32, 33). Additionally, chronic inflammation, such as exposure to IFNα (34) and oxidative stress (32), may contribute to DDR activation. Of note, oxidative stress in SLE causes mitochondrial hyperpolarization and ATP depletion, leading to necrotic cell death, especially in T cells, which further releases oxidized DNA (35, 36). Thus, impaired DNA damage repair, exposure to IFNα and oxidative stress may induce IFNα production by monocytes. However, further studies are needed to fully understand these mechanisms.

GATA4 is a member of the GATA family of zinc-finger transcription factors and is important for tissue homeostasis and the development of organs such as the heart, lung, liver, and pancreas (37). GATA4 is also a critical regulator of SASP, which modulates the expressions of key cytokines such as IL-1α, IL-6, and IL-8 through NF-κB signaling (16) in senescent cells. In our study, GATA4 was upregulated in IFNα-producing SLE monocytes and overexpression experiments confirmed that GATA4 positively regulated cGAS-STING-mediated IFNα induction, indicating that GATA4 also regulates IFNα induction.

Although GATA4 expression was not detected in the unstimulated SLE monocytes in our study or in publicly available RNA-seq data, a study re-analyzing publicly available transcriptome data of PBMCs from HC and SLE identified GATA4 as a transcription factor with inferred higher activity compared with other transcription factors (38). This suggested that GATA4 is involved in lupus pathogenesis. We hypothesized that GATA4 might bind to the promoter regions of IFNA genes. However, no enrichment of GATA4 binding was detected within any of these genes (data not shown). We further identified that IFIT genes are directly regulated by GATA4, which binds to the enhancer region of IFIT genes, which are involved in type I IFN production. IFIT1 was shown to enhance the induction of type I IFNs by promoting the translocation of phosphorylated IRF3 into the nucleus (17). IFIT3 enhances the binding of TBK1 to STING, resulting in the increased production of IFNβ (18). Regarding SLE, IFIT genes were upregulated in the monocytes of SLE patients (39). Thus, the expression of GATA4 in SLE monocytes leads to the upregulation of IFIT genes and the subsequent induction of type I IFNs.

**Figure 6:**
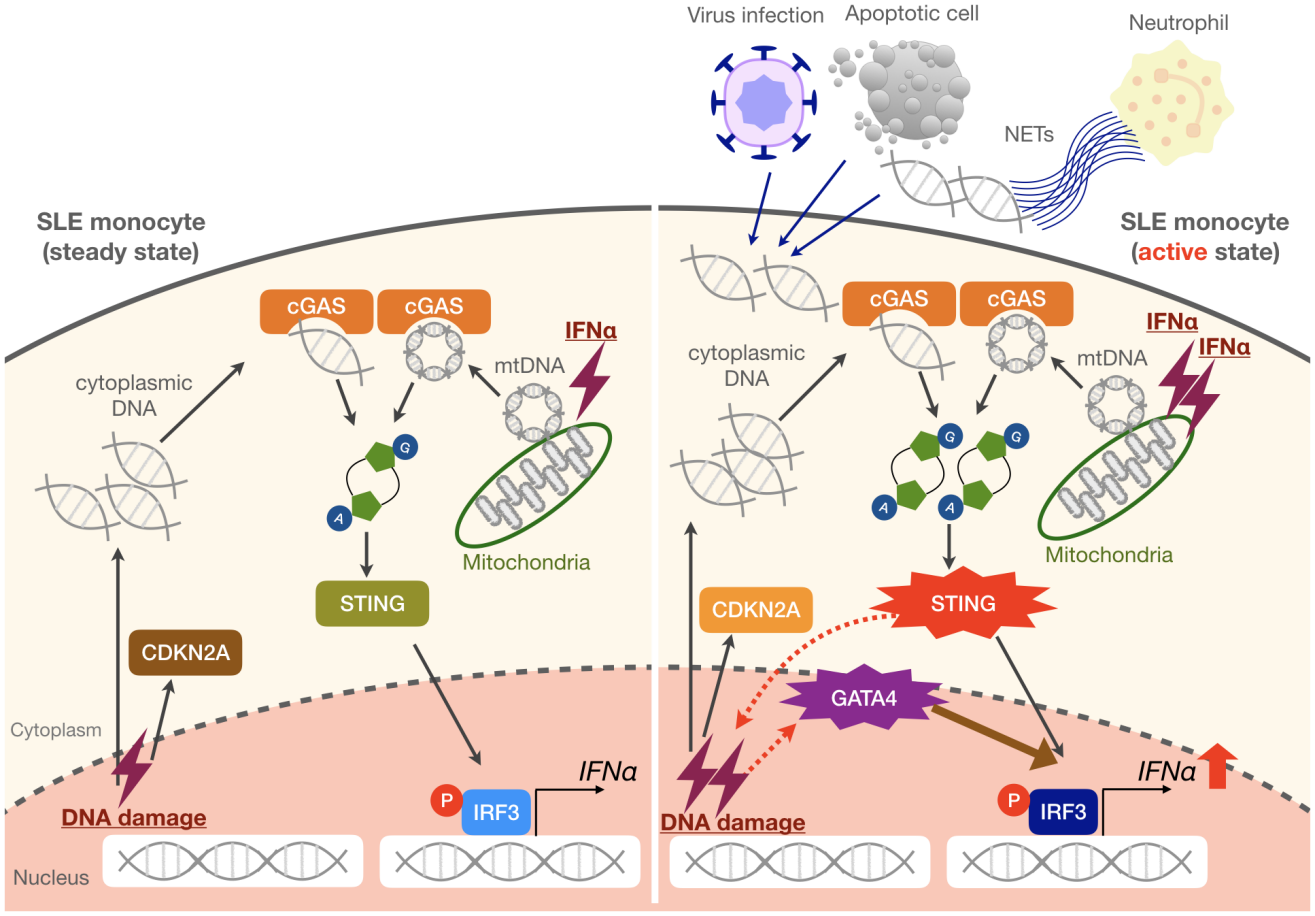
Proposed mechanisms of enhanced IFNα production in SLE monocytes. Steady-state SLE monocytes exhibit a cellular senescence phenotype as evidenced by CDKN2A upregulation. When exposed to large amounts of self-DNA derived from various triggers such as viruses, apoptotic cells, and NETs, as well as increased mitochondrial DNA (mtDNA) in the cytoplasm, the activation of the cGAS-STING pathway is enhanced in SLE monocytes. This results in the promotion of cellular senescence and GATA4 induction, which further enhances IFNα production. We propose that these mechanisms may be responsible for a vicious cycle of inflammation, especially during an active disease state or flare in SLE patients.

Although the pathogenic role of monocytes in SLE remains unclear, they were reported to infiltrate inflammatory sites. Recently, the single-cell RNA-sequencing of kidney and blood samples from patients with lupus nephritis revealed monocyte-derived macrophage clusters were expanded in SLE (40). In our previous report using imiquimod-induced lupus mice, we showed that numbers of Ly6C^lo^ monocytes with enhanced inflammatory potential were increased in the peripheral blood and were able to infiltrate into the kidneys and replace resident macrophages (41). These results suggest that lupus monocytes are already activated in the peripheral blood and exert their functions in the inflamed lesions. Although IFNα levels are elevated in the serum of SLE patients, a previous single-cell RNA-seq analysis of PBMCs from pediatric and adult SLE patients did not detect the expressions of type I IFNs in any cell subsets, including pDCs (42). These findings suggest that detectable levels of type I IFNs produced by myeloid cells are mainly triggered in inflamed tissues or secondary lymphoid organs and are related to the exposure to self-DNA derived from damaged cells, apoptotic cells, and NETs. Such exposure can lead to IFNα production by pDCs via TLR7 and by monocytes via cGAS-STING, both of which contribute to disease exacerbation. In monocytes, increased cGAS-STING activation by sensing cytoplasmic DNA can also lead to the promotion of cellular senescence and induction of GATA4, which further increases the production of IFNα (**Figure 6**). Interestingly, a recent study demonstrated that impaired mitophagy led to the non-removal of mitochondria from red blood cells in some SLE patients, which promoted the induction of type I IFNs via cGAS-STING when phagocytosed by monocyte-derived macrophages *in vitro* (43), suggesting another mechanism that can activate cGAS-STING in monocytic cells.

In conclusion, we showed that SLE monocytes exhibit a cellular senescence phenotype, resulting in the expression of GATA4 and enhanced IFNα production by activation of the STING pathway. Thus, targeting cellular senescence may be beneficial for the treatment of SLE.

## Data availability statement

The datasets presented in this study can be found in online repositories. The names of the repository/repositories and accession number(s) can be found in the article/**Supplementary Material**. The RNA-seq data and the ChIP-seq data were deposited in the DNA Data Bank of Japan (DDBJ) under BioProject ID PRJDB16438 and PRJDB16394 https://www.ncbi.nlm.nih.gov/bioproject/?term=PRJDB16438, https://www.ncbi.nlm.nih.gov/bioproject/?term=PRJDB16394.

## Ethics statement

The studies involving humans were approved by ethics committee at Juntendo University Hospital. The studies were conducted in accordance with the local legislation and institutional requirements. The participants provided their written informed consent to participate in this study.

## Author contributions

TK: Formal analysis, Investigation, Methodology, Writing – original draft, Writing – review & editing. AC: Conceptualization, Funding acquisition, Supervision, Validation, Writing – original draft, Writing – review & editing. GM: Conceptualization, Funding acquisition, Methodology, Resources, Writing – original draft, Writing – review & editing. KH: Investigation, Writing – original draft. TN: Investigation, Writing – original draft. YY: Investigation, Methodology, Writing – review & editing. DN: Methodology, Validation, Writing – original draft. MK: Funding acquisition, Resources, Writing – original draft. FK: Methodology, Validation, Writing – original draft, Writing – review & editing. KY: Funding acquisition, Resources, Writing – original draft. NT: Funding acquisition, Supervision, Writing – original draft, Writing – review & editing. SM: Conceptualization, Funding acquisition, Supervision, Validation, Writing – original draft, Writing – review & editing.
