# Supplementary material for: Enhanced GATA4 expression in senescent systemic lupus erythematosus monocytes promotes high levels of IFNα production
Source: Front Immunol. 2024 Mar 28;15:1320444. doi: 10.3389/fimmu.2024.1320444 (PMC11007064; doi:10.3389/fimmu.2024.1320444)
Supplement: Supplementary file 1 [file DataSheet_1.pdf]

Supplementary Figure S1.

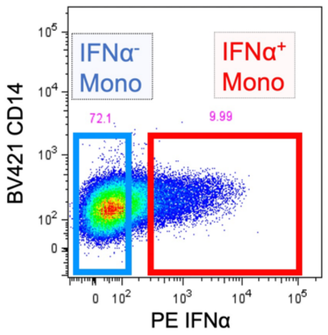


**Supplementary Figure S1. Gating strategy for FACS sorting of IFNα positive/negative monocytes for RNA-seq analysis.**

$CD14^+$ cells enriched from PBMCs were stimulated with 2'3'-cGAMP for 5 h, and secretion assays were performed to detect IFNα producing cells. Monocytes were gated using a forward scatter area (FSC-A) vs side scatter area (SSC-A) plot. Single cells were selected using FSC-A vs FSC-height (FSC-H) and SSC-A vs SSC-height (SSC-H) plots. 7-AAD positive dead cells, $CD3^+$ T cells, $CD19^+$ B cells, $CD56^+$ NK cells, CD123 high plasmacytoid dendritic cells, and CD11c high dendritic cells were excluded. IFNα-positive and IFNα-negative monocytes were gated as shown and sorted using FACS Aria III.

Supplementary Figure S2.

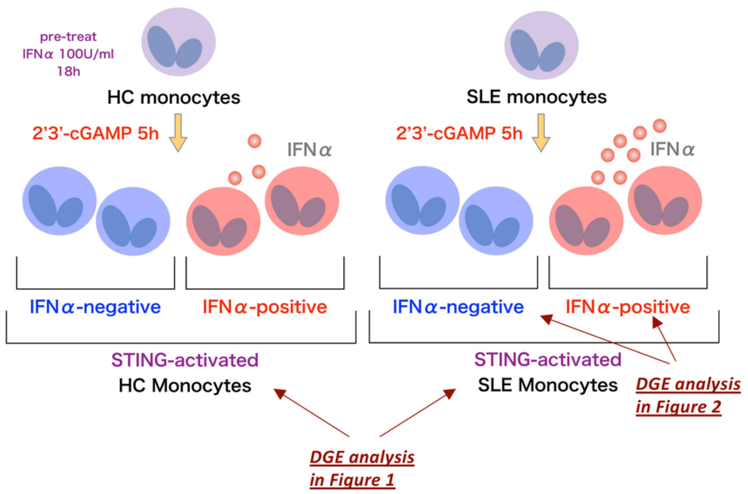


**Supplementary Figure S2. Experimental design of the RNA-seq analysis of STING-activated monocytes.**

A schematic diagram of the experimental design and groups for differential gene expression (DGE) analysis in Figures 1 and 2. In Figure 1, we combined IFNα-positive and IFNα-negative monocytes and compared the transcriptional profiles of STING-activated SLE and HC monocyte, and in Figure 2, we compared the transcriptional profiles of IFNα-positive SLE monocytes and IFNα-negative SLE monocytes.

Supplementary Figure S3.

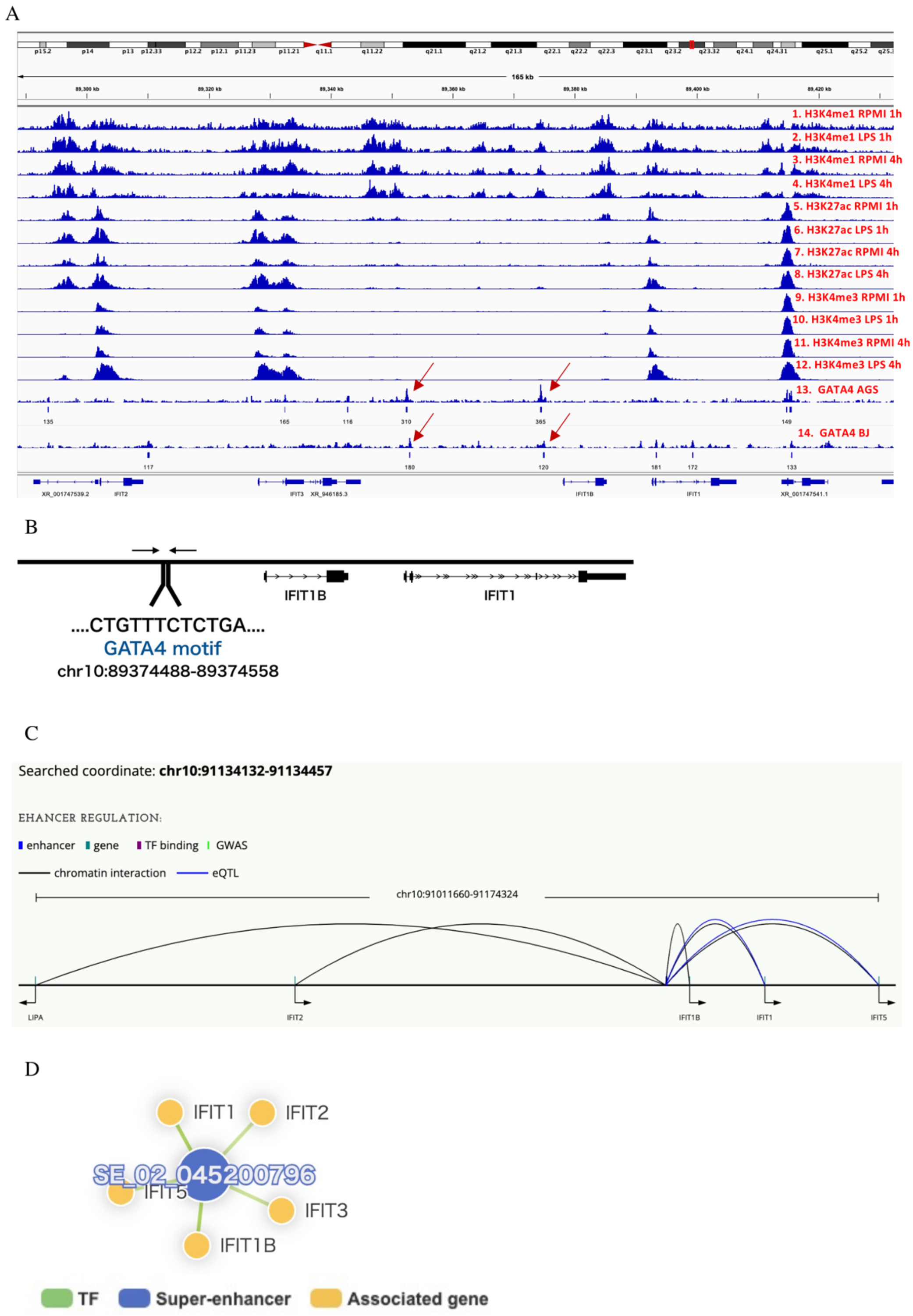

**Supplementary Figure S3. GATA4 binding sites in the genomic region of IFIT genes.**
(A) A genome browser image of the genomic regions surrounding IFIT1B and IFIT1, displaying the histone modification status. The data were processed and obtained from the ChIP-Atlas database, which analyzes publicly-available ChIP-seq datasets. The red arrows indicate predicted GATA4-binding sites. Datasets used are listed in Supplemental table S4.
(B) A schematic representation of the genomic regions of IFIT genes with the indicated qPCR primers. Candidate GATA4 motifs were identified using the JASPER database (https://jaspar.genereg.net/) with a relative profile score threshold > 70%, and a primer pair spanning the motif was designed. The primer sequence is as follows: forward, 5′-GGACTGCTGCCAAATTCAGTG-3′; reverse, 5′-TGCTAGTAACAGCGTGTCAG-3′.
(C) The GATA4 binding region (chr10:91134132-91134457; GRCh37/hg 19 assembly) was searched for in the HACER database (https://bioinfo.vanderbilt.edu/AE/HACER/index.html).
(D) The GATA4 binding region (chr10:89374325-89374685; GRCh38/hg38 assembly) was searched for in the human super-enhancers database SEdb2.0 (https://bio.liclab.net/sedb/index.php). The database showed that the region was included in the super-enhancer (SE_02_045200796), which regulates the expressions of IFIT genes including IFIT1, IFIT2, and IFIT3.

Supplementary Figure S4

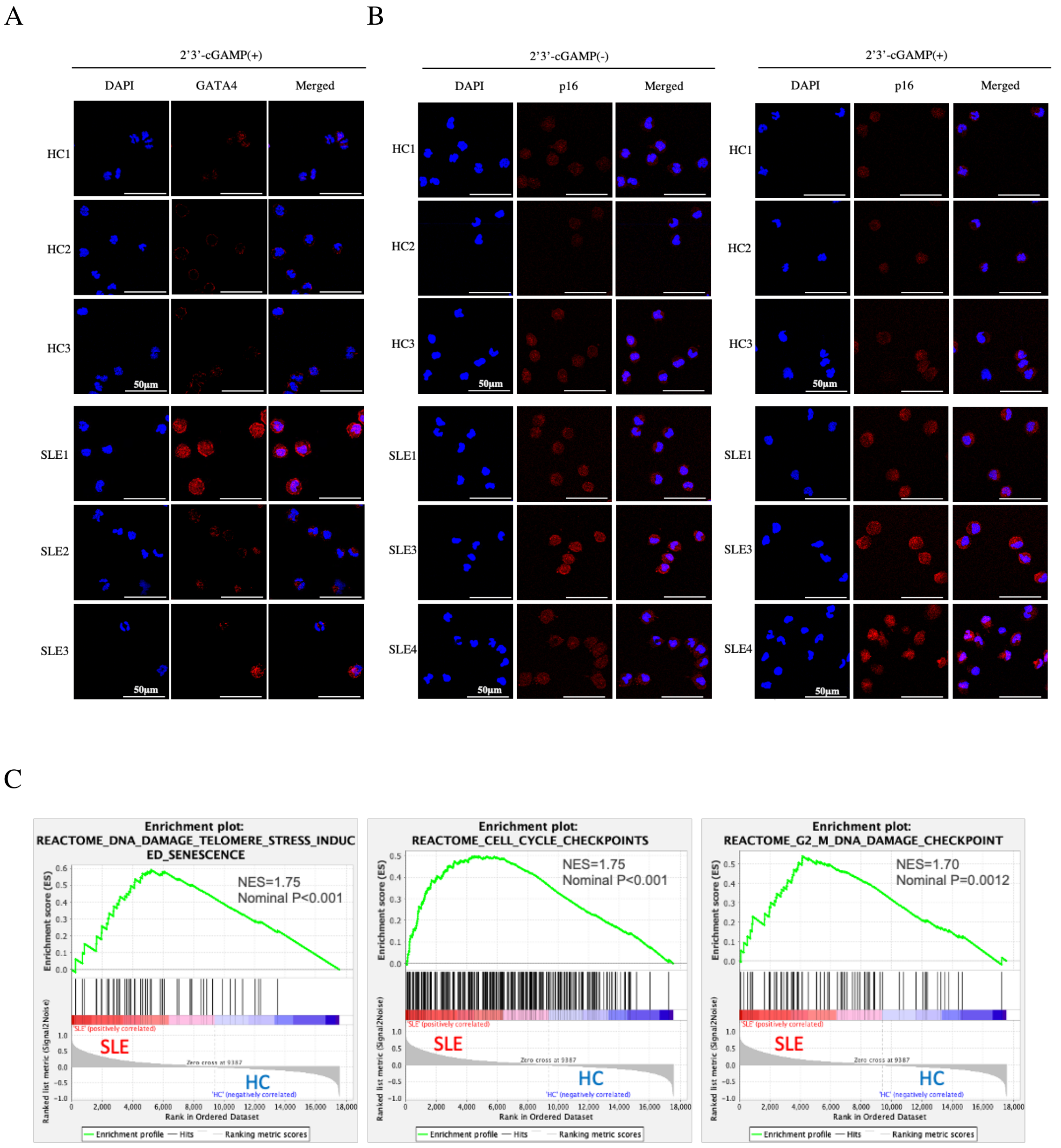


**Supplementary Figure S4. Cellular senescence-like phenotype was observed in SLE monocytes.**

(A) Immunofluorescence images showing GATA4 (red) staining of monocytes stimulated with 2′3′-cGAMP. Cell nuclei were stained with DAPI. Representative images from three HC and three SLE patients are shown. (B) Immunofluorescence images showing p16 (red) staining of monocytes, with or without 2′3′-cGAMP stimulation. Cell nuclei were stained with DAPI. Representative images from HC and SLE patients are presented. (C) GSEA of steady-state SLE and HC monocytes using the

publicly available RNA-seq dataset (BioProject PRJNA:392602). GSEA plot showing the enrichment of “DNA Damage Telomere Stress Induced Senescence (R-HSA-2559586)”, “Cell Cycle Check Points (R-HSA-69620)”, and “G2 M DNA Damage Check Point (R-HSA-69473)” in SLE steady-state monocytes.
