# Supplementary material for: Enhanced GATA4 expression in senescent systemic lupus erythematosus monocytes promotes high levels of IFNα production
Source: Front Immunol. 2024 Mar 28;15:1320444. doi: 10.3389/fimmu.2024.1320444 (PMC11007064; doi:10.3389/fimmu.2024.1320444)
Supplement: Supplementary file 2 [file DataSheet_2.pdf]

Supplementary table S1.

Characteristics of HC and patients with SLE in the RNA-seq analysis

	Healthy controls	SLE
Number, n	5	5
Females/males, n	3/2	3/2
Age, years, median (IQR)	33.0 (28.5-52.5)	30.0 (28.5-50.0)
Disease duration, years, median (IQR)	-	1.8 (0.05-19.5)
Medications		
Medication naïve, n	-	2
Predonisone, n	-	3
Prednisone dose, mg/day, median (IQR)	-	11.0 (10.0-20.0)
HCQ, n	-	1
Immunosuppressive agent[a] , n	-	2

IQR, interquartile range

[a]mycophenolate mofetil, tacrolimus, azathioprine

Supplementary table S2.

Characteristics of HC and patients with SLE in the RT-PCR analysis

	Healthy controls	SLE
Number, n	6	10
Females/males, n	5/1	9/1
Age, years, median (IQR)	37.5 (30.5-48.8)	45.5 (35.3-50.0)
Disease duration, years, median (IQR)	-	17 (4.0-24.0)
Medications		
Medication naïve, n	-	1
Predonisone, n	-	9
Prednisone dose, mg/day, median (IQR)	-	7.75 (7.0-8.0)
HCQ, n	-	5
Immunosuppressive agent[a] , n	-	4

IQR, interquartile range

[a]mycophenolate mofetil, tacrolimus

Supplementary table S3.

Characteristics of HC and patients with SLE in SA-β-Gal analysis

	Healthy controls	SLE
Number, n	6	10
Females/males, n	5/1	8/2
Age, years, median (IQR)	42 (31.3-53.3)	47 (37.0-60.0)
Disease duration, years, median (IQR)	-	1.8 (0.05-19.5)
Medications		
Medication naïve, n	-	0
Predonisone, n	-	10
Prednisone dose, mg/day, median (IQR)	-	4.8(3.4-5.8)
HCQ, n	-	3
Immunosuppressive agent[a] , n	-	7

IQR, interquartile range

[a]mycophenolate mofetil, tacrolimus, azathioprine

Supplementary table S4.

Primers used in this study for mRNA quantification.

Name	Forward	Reverse
GAPDH	CAGTCAGCCGCATCTTCTTTTG	GCGCCCAATACGACCAAAT
CDKN2A	GAAGGTCCCTCAGACATCCC	GGACCTTCGGTGACTGATGA
GATA4	GTCATCTCACTACGGGCACA	TAGCCTTGTGGGGAGAGCTT
IFNA1	GGAGGAAGGAATAACATCTGGTCC	GCAGGGGTGAGAGTCTTTGAA
IFNB1	GCTCTGGCACAACAGGTAG	TAGTGGAGAAGCACAACAGG
IFIT1	GCCTCCTTGGGTTCGTCTATAA	TCAAAGTCAGCAGCCAGTCTCA
IFIT2	CACTGCAACCATGAGTGAGAACA	AGATAGGCCAGTAGGTTGCAC
IFIT3	GGCAGTCATGAGTGAGGTC	TGAATAAGTTCCAGGTGAAATGGC

Supplementary table S5.

Publicly-available ChIP-seq datasets, which were processed and obtained from the ChIP-Atlas database in Supplementary figure S3A.

	Accession No.	Sample	Cell	ChIP_antibody	Source_publication
1	GSM2262943	RPMI_1h_rep1_H3K4me1	Monocyte	H3K4me1	Novakovic, et al. Cell 167, 1354−1368 (2016)
2	GSM2262941	LPS_1h_rep1_H3K4me1	Monocyte	H3K4me1	Novakovic, et al. Cell 167, 1354−1368 (2016)
3	GSM2262948	RPMI_4h_rep1_H3K4me1	Monocyte	H3K4me1	Novakovic, et al. Cell 167, 1354−1368 (2016)
4	GSM2263049	LPS_4h_rep1_H3K4me1	Monocyte	H3K4me1	Novakovic, et al. Cell 167, 1354−1368 (2016)
5	GSM2263026	RPMI_1h_rep1_H3K27ac	Monocyte	H3K27ac	Novakovic, et al. Cell 167, 1354−1368 (2016)
6	GSM2262923	LPS_1h_rep1_H3K27ac	Monocyte	H3K27ac	Novakovic, et al. Cell 167, 1354−1368 (2016)
7	GSM2263019	RPMI_4h_rep1_H3K27ac	Monocyte	H3K27ac	Novakovic, et al. Cell 167, 1354−1368 (2016)
8	GSM2263011	LPS_4h_rep1_H3K27ac	Monocyte	H3K27ac	Novakovic, et al. Cell 167, 1354−1368 (2016)
9	GSM2262926	RPMI_1h_rep1_H3K4me3	Monocyte	H3K4me3	Novakovic, et al. Cell 167, 1354−1368 (2016)
10	GSM2262987	LPS_1h_rep1_H3K4me3	Monocyte	H3K4me3	Novakovic, et al. Cell 167, 1354−1368 (2016)
11	GSM2262976	RPMI_4h_rep1_H3K4me3	Monocyte	H3K4me3	Novakovic, et al. Cell 167, 1354−1368 (2016)
12	GSM2263038	LPS_4h_rep1_H3K4me3	Monocyte	H3K4me3	Novakovic, et al. Cell 167, 1354−1368 (2016)
13	GSM1250896	AGS GATA4	AGS cellline	GATA4	Chia N, Deng N, Das K, et al. Gut 64, 707-719 (2015)
14	GSM2430676	GATA4 ChIP	BJFOXA2 line	GATA4	Donaghey J, Thakurela S, Charlton J, Chen JS et al. Nat Genet 50, 250-258 (2018)
